# Solubility of Two Resin Composites in Different Mouthrinses

**DOI:** 10.1155/2014/580675

**Published:** 2014-04-07

**Authors:** Sezin Ozer, Emine Sen Tunc, Nuray Tuloglu, Sule Bayrak

**Affiliations:** ^1^Faculty of Dentistry, Department of Pediatric Dentistry, Ondokuz Mayıs University, Kurupelit, 55139 Samsun, Turkey; ^2^Department of Pediatric Dentistry, Faculty of Dentistry, Eskisehir Osmangazi University, Eskisehir, Turkey

## Abstract

*Aim*. This study aimed to compare the solubility of a universal restorative resin composite (Filtek Z250; FZ250) and a silorane-based resin composite (Filtek Silorane; FS) after immersion in alcohol-containing mouthrinse, alcohol-free mouthrinse, and artificial saliva. *Methods*. 30 discs (10 mm × 1 mm) were prepared from each material and desiccated until a constant mass was obtained. Specimens were immersed in the test solutions for two days and desiccated again. Solubility was calculated based on the change in weight of each specimen before and after immersion. Data was analyzed using two-way ANOVA and Tukey's Post Hoc test (*P* < 0.05). *Results*. Solubility values for both resin composites were the highest in the alcohol-containing mouthrinse. FZ250 showed greater solubility than FS; the difference was only significant in artificial saliva. *Conclusion*. Both resin-composite materials tested exhibited some degree of solubility in each of the test solutions. The use of an alcohol-free mouthrinse may be preferable for patients with extensive composite restorations.

## 1. Introduction

Dental composites constitute an important group of materials in modern restorative dentistry [1]. Composites—which generally consist of a resin matrix, inorganic filler, and coupling agent—are becoming more and more popular as dental restorative materials because of their strength, rapid polymerization, and esthetic appearance [2]. However, polymerization shrinkage and shrinkage-related stress are two major drawbacks of resin composites that still need to be addressed. In order to overcome these shortcomings, dental siloranes consisting of a new type of organic matrix (i.e., monomers with a ring-opening oxirane) were introduced in 2007 [3].

Upon intraoral application, resin composites face constant exposure to an aqueous environment. Water diffused into the resin matrix may contribute to the relaxation of polymerization shrinkage stress to some extent [4], and, by expanding the polymer matrix and increasing the bulk volume of the resin composite, water sorption may reduce the size of marginal gaps generated by polymerization shrinkage [5]. On the other hand, water sorption may lead to degradation of the resin matrix and debonding of the matrix-filler interface, resulting in a deterioration of mechanical properties. In addition, leakage of fillers, ions, and organic substances such as residual monomers, methacrylic acid, and formaldehyde from resin composite material can occur as a result of exposure to an aqueous environment. Some of these organic substances are potent irritants and may induce delayed allergic reactions. Both material degradation and leakage have been shown to be dependent upon time and material composition [6].

Effective plaque control is crucial for the maintenance of periodontal health and control of cariogenic activity. For most individuals, toothbrushing with a dentifrice is the most efficient, safe and economical method of removing plaque; however, many individuals find it difficult to achieve a plaque-free dentition with this method alone. While the use of an antiseptic agent such as a mouthrinse as an adjunct to toothbrushing with a dentifrice may be justified, especially in caries-active patients [7, 8], the detrimental effects of mouthrinses on resin composite restorations must be taken into account [9].

The organic matrix of conventional resin composites is generally based on methacrylate chemistry, especially cross-linking dimethacrylates. The solubility of dimethacrylate-based resin composites in various solutions including water and mouthrinses has been widely studied [2, 10–12]; however, few studies have been carried out on silorane-based resin composites [13–15]. Therefore, this study aimed to measure the solubility of two different resin composites in three different solutions: an alcohol-free mouthrinse, an alcohol-containing mouthrinse, and artificial saliva [16]. The null hypothesis was that solubility values would not differ, regardless of the restorative material or solution tested.

## 2. Materials and Methods

### 2.1. Restorative Materials and Solutions

This study employed a randomized factorial design conducted with 10 replications of 2 different restorative materials (FZ250 and FS) and 3 different solutions (artificial saliva, an alcohol-free mouthrinse (Oral-B, Pro-Expert), and an alcohol-containing mouthrinse (Oral-B, Advantage)) (Table 1).

### 2.2. Preparation of Specimens

For each material, a total of 30 disc-shaped specimens were prepared using a plastic mold (1 ± 0.1 mm × 10 ± 0.1 mm). The mold was filled and pressed by hand between two glass microscope slides to extrude any excess material. Specimens were light-cured on both sides and in different positions for a total of 60 seconds using an LED curing unit (Elipar Free Light II, 3 M/ESPE, St. Paul, MN, USA; light intensity: 1000 mV/cm^2^). Specimens were removed from the molds, and any excess material was removed by gently grinding on both sides with 600-grit silicon carbide paper (Phoenix Beta, Buehler, Germany). Debris was removed with a dust blower.

### 2.3. Solubility Measurements

Solubility measurements were conducted in line with ISO 4049 standards [17]. A digital caliper (Digimatic Caliper, Mitutoyo, Tokyo, Japan; accuracy = 0.002 mm) was used to measure diameter and thickness. The diameter of each specimen was measured at two points at right angles to one another, and the mean diameter was calculated. The thickness of each specimen was measured at the center of the specimen and at four equally spaced points on the circumference, and the mean thickness was calculated. The volume (*V*) of each specimen was calculated in mm^3^ using the formula *V* = *π* × *r* ^2^ × *h*, where *r* is the mean sample radius (diameter/2) and *h* is the mean sample thickness.

For each material, 10 specimens were placed in a glass vial containing 10 mL of artificial saliva, 10 in a glass vial containing 10 mL of alcohol-free mouthrinse (Oral-B, Pro-Expert) and 10 in a glass vial containing 10 mL of alcohol-containing mouthrinse (Oral-B, Advantage). Vials were wrapped in aluminum foil to exclude light and placed in an incubator at 37°C.

Specimens were weighed using an analytical scale accurate up to 0.0001 mg (Precise XB 220A, Switzerland). All specimens were stored in a vacuum desiccator at 37°C until a constant weight (*m*_0_) was obtained. Specimens were then immersed in the solutions for 2 days, removed, and desiccated again until a constant mass was obtained, and the weights were recorded again (*m*_1_). Solubility (SL) was recorded in *μ*g/mm^3^ as the change in weight before and after immersion using the formula SL = *m*_0_ − *m*_1_/*V*, where *V*  is the volume of the sample in mm^3^.

### 2.4. Statistical Analysis

Statistical analysis was conducted using the software SPSS 12.0 (SPSS Software, Munich, Germany). Means and standard deviations were calculated, and multiple comparisons were performed using two-way ANOVA and Tukey's Post Hoc test, with a level of *P* < 0.05 considered statistically significant.

## 3. Results

Means, standard deviations, and significant differences in solubility are presented in Table 2. Solubility values of FS were lower than those of FZ250 in each of the solutions tested; however, these differences were only statistically significant in artificial saliva (*P* < 0.05). Although no statistically significant differences were observed between the solubility values of the same material in different mouthrinses (*P* > 0.05), solubility values for both materials were higher in the alcohol-containing mouthrinse than in the alcohol-free mouthrinse.

## 4. Discussion

The current study found the solubility of the universal resin composite FZ250 to be higher than that of the silorane resin composite FS; however, the difference between the two was only statistically significant in artificial saliva (*P* < 0.05). Thus, the null hypothesis was accepted, with the exception of solubility performance in artificial saliva.

Excessive solubility of dental restorative materials can lead to surface deformation and marginal discrepancies. Resin composite materials come into extensive contact with oral fluids, food components, and drinks in the oral environment [18], and while the solubility of dimethacrylate-based resin composites in different solutions has been widely studied [2, 10–12], to our knowledge, there is very little information available regarding the solubility of silorane-based resin composites [13–15]. Therefore, the present study aimed to evaluate the solubility of FS and FZ250 in 3 different solutions.

Solubility mean values presented by the composite resins tested varied from 2.3 to 4.2 *μ*g/mm^3^; these values were lower than the maximum value established by the ISO 4049 standard (<7.5 *μ*g/mm^3^) [17]. Although the direct exploration is not possible, the results of the present study showed that the solubility of all materials in all solutions is acceptable for ISO 4049.

With the exception of artificial saliva, the solubility of FS and FZ250 did not differ significantly (*P* > 0.05); however, the solubility of FZ250 was greater than that of FS. It is likely that the low solubility of FS is related to the distinctive polymerization characteristics of the material. This finding is consistent with a previous study showing silorane-based resin composites to be more hydrophobic than methacrylate-based resin composites [13, 19]. The solubility of resin composites is related to the dissolution and leaching of various components, particularly unreacted monomers [20]. The organic matrix of conventional resin composites is generally based on methacrylate chemistry, especially cross-linking methacrylates, as in FZ250. The density of the links in methacrylate-based resin composites may vary as a result of the polymerization of free radicals, causing spatial heterogeneity that may facilitate the entrapment of residual monomers in microgels, from where they may be easily leached [21]. When compared to methacrylate-based resin polymerization, the photoactivated cationic polymerization process of silorane resins is relatively insensitive to oxygen. Not only does this reduce polymerization shrinkage, it also increases the degree of conversion [15, 22, 23].

Ethanol, which is found in many mouthrinses, may accelerate the hydrolytic degradation of resin-based materials [24]. In vitro studies have reproduced the subsurface and surface degradation of resin composites by storing them in ethanol [6], and the mechanical properties of composite resins have been compromised by aging them in alcohol-containing solutions [9]. In the present study, both materials tested exhibited greater solubility in the mouthrinse containing alcohol when compared to the alcohol-free mouthrinse (*P* > 0.05). These findings suggest that it may be preferable for patients with extensive restorations to avoid the use of mouthrinse containing alcohol as part of their daily oral hygiene routine so as to prevent the need for recurrent restorative treatment.

As with all in vitro studies, caution must be used when extrapolating the results of the present study to the oral environment. Clinical studies are needed to examine the in vivo solubility behavior of different resin composites.

## 5. Conclusion

Within the limitations of this in vitro study, the following conclusions can be drawn:

1. the solubility of FS was lower than that of FZ250 in all the solutions tested;
2. solubility of both of the restorative materials tested was lower in alcohol-free mouthrinse than in alcohol mouthrinse containing;
3. alcohol-free mouthrinse may prefer to alcohol containing mouthrinse in patients with extensive restorations.

## Figures and Tables

**Table 1:** Materials used in the study.

Material	Contents	Lot no	Manufacturer
Filtek Z250 universal restorative	Bisphenol A polyethyleneglycol diether dimethacrylate (bis-EMA) (5–10% by wt), silane treated ceramic (75–85% by wt), urethane dimethacrylate (UDMA) (5–10% by wt), bisphenol-A-glycidyl dimethacrylate (Bis-GMA) (<5% by wt), triethyleneglycol dimethacrylate (TEGDMA) (<5% by wt), and water Filled to 60% by volume with zircon silica filler, average particle size = 0.6 µm	8FA	3M/ESPE, St. Paul, MN, USA

Filtek Silorane (posterior restorative)	Silorane (3,4-epoxycyclohexylethylcyclo-polymethylsiloxane, bis-3,4-epoxycyclohexylethyl-phenylmethylsilane) Fillers: Quarz (silane layer) radiopaque yttrium fluoride Filler loading 76% (wt %)	N236344	3M/ESPE, St. Paul, MN, USA

Oral-B, Pro-Expert mouthrinse (alcohol-free mouthrinse)	Aqua, Glycerin, polysorbate 20, Aroma, methylparaben, cetylpyridinium chloride, sodium fluoride, sodium saccharin, sodium benzoate, propylparaben, Cl 42051, and Cl 47005	99602155	Procter & Gamble MN GmbH, Stra*β*e e 1, 64521 Gross Gerau, Germany

Oral-B, Advantage mouthrinse (alcohol-containing mouthrinse)	Aqua, glycerin, alcohol, aroma, methylparaben, poloxamer 407, cetylpyridinium chloride, sodium fluoride, sodium saccharin, Propylparaben, Cl 42051, and Cl 47005	95587215	Procter & Gamble UK, Weybrige, KT13 0XP

Artificial saliva	NaCl (400 mg/L), KCL (400 mg/L), CaCl_2_·2H_2_O (795 mg/L), NaH_2_PO_4_·H_2_O (690 mg/L) KSCN (300 mg/L), Na_2_S·9H_2_O (5 mg/L), and urea (1000 mg/L)		

**Table 2:** Mean solubility values of materials tested (µg/mm^3^).

Solutions	Test materials	
	FZ250 (sd)	FS (sd)
Alcohol-containing mouth rinse (advantage)	4.2 (1,4)^(A,1)^	3.1 (1,7)^(B,1)^
Alcohol-free mouth rinse (Pro-Expert)	3.5 (0,9)^(A,1)^	3.0 (1,1)^(B,1)^
Artificial saliva	3.4 (1,3)^(A,1)^	2.3 (0,6)^(B,2)^

Differences in superscript letters indicate statistically significant differences within columns and differences in superscript numbers indicate significant differences within rows.
